# Spin-orbit coupling in van der Waals materials for optical vortex generation

**DOI:** 10.1038/s41377-025-01926-7

**Published:** 2025-08-18

**Authors:** Jaegang Jo, Sujeong Byun, Munseong Bae, Jianwei Wang, Haejun Chung, Sejeong Kim

**Affiliations:** 1 Department of Electronic Engineering, Hanyang University, Seoul, Republic of Korea; 2 Department of Electrical and Electronic Engineering, Faculty of Engineering and Information Technology, University of Melbourne, Melbourne, Australia; 3 State Key Laboratory for Mesoscopic Physics and Collaborative Innovation Center of Quantum Matter, School of Physics, Peking University, Beijing, China

**Keywords:** Optical materials and structures, Optical physics

## Abstract

An optical vortex beam has attracted significant attention across diverse applications, including optical manipulation, phase-contrast microscopy, optical communication, and quantum photonics. To utilize vortex generators for integrated photonics, researchers have developed ultra-compact vortex generators using fork gratings, metasurfaces, and integrated microcombs. However, those devices depend on costly, time-consuming nanofabrication and are constrained by the low signal-to-noise ratio due to the fabrication error. As an alternative maneuver, spin-orbit coupling has emerged as a method to obtain the vortex beam by converting spin angular momentum (SAM) without nanostructures. Here, we demonstrate the creation of an optical vortex beam using van der Waals (vdW) materials. The significantly high birefringence of vdW materials allows the generation of optical vortex beams, even with materials of sub-wavelength thickness. In this work, we utilize an 8 µm-thick hexagonal boron nitride (hBN) crystal for the creation of optical vortices carrying topological charges of ±2. We also present the generation of an optical vortex beam in a 320 nm-thick MoS_2_ crystal with a conversion efficiency of 0.09. This study paves the way for fabrication-less and ultra-compact optical vortex generators, which can be applied for integrated photonics and large-scale vortex generator arrays.

## Abstract (web summary)

Once the focused circularly polarized light propagates the van der Waals crystal, the optical vortex beam with topological charge of $\pm$2 is generated due to the spin-orbit coupling.

## Graphical abstract

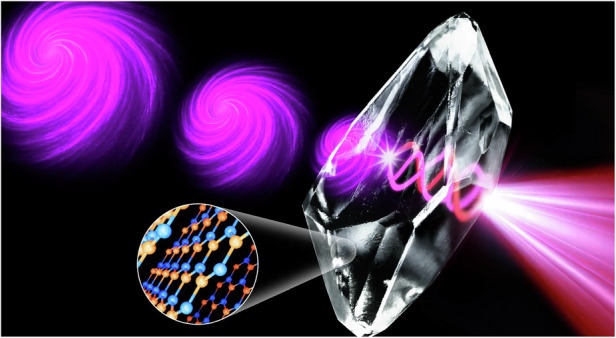

## Introduction

Over the past decades, optical vortex beams, characterized by their orbital angular momentum (OAM), have been extensively investigated for various applications, including optical micro-manipulation^1–4^, chirality sensing^5–7^, and phase contrast microscopy^8,9^. The topological charges, defined by the number of 2*π* phase shifts along the beam’s axis, allow an enhanced degree of freedom and increase data capacity for communication^10–12^. Optical vortex beams can also be used to multiplex the states of qubits and realize OAM qubits, opening exciting research avenues in quantum optics^13–16^. Upon the introduction of the concept of optical vortices in 1989^17^, extensive studies have been dedicated to developing methods for creating light sources with OAM. Over the years, various commercially available solutions have successfully demonstrated tools for generating optical vortices with various topological charges. These solutions include spiral phase plates^18,19^, q-plates^20^, and computer-generated holograms, also known as spatial light modulators^21,22^. While these methods are reliable in producing vortex beams, they often involve bulky optical systems. Consequently, there has been significant interest in advancing more compact and integrated photonics solutions for optical vortex generation. Early approaches involve the use of liquid crystals, which offer some level of integration^23^. Recent advancements have introduced the use of fork gratings^24,25^, metasurfaces^26–29^, integrated microcombs^30–33^, and inverse-designed devices^11,34^, which are capable of generating optical vortices on a chip. However, existing on-chip solutions involve nano-scale features that require costly, time-consuming nanofabrications and often encounter challenges in achieving sufficient signal-to-noise ratios.

While previously reported on-chip vortex generation methods usually involve phase-front manipulation to convert non-structured light into helically structured light, an emerging solution reveals the new possibility of creating optical vortices by harnessing optical spin-orbit coupling^35,36^. A theoretical proof in the previous study^37^ demonstrated that the circularly polarized light (CPL) beam incident on any uniaxial medium gives rise to the light with the opposite handedness, thus imparting OAM to the transmitted beam. Recently, this theoretical concept was experimentally validated using lithium niobate (LN)^38^ and beta-barium borate (BBO) crystals^14,15^. However, such materials have small birefringence (0.09 and 0.12 at *λ* = 594 nm for LN^39^, and BBO crystals^40^, respectively); thus, the vortex generators made from these crystals necessitate bulky materials, often exceeding several millimeters in thickness to achieve sufficient conversion efficiencies^41^.

Here, we propose and demonstrate a fabrication-free optical vortex generator based on van der Waals (vdW) materials. By leveraging the giant birefringence in vdW materials, we induce efficient spin-orbit coupling within the medium to generate an optical vortex beam, leading to a thin optical vortex generator. For this demonstration, we selected hexagonal boron nitride (hBN) and molybdenum disulfide (MoS_2_) among various vdW materials due to their high birefringence. hBN is transparent across a wide wavelength range, from visible to infrared, while exhibiting high birefringence (*n*_*o*_ ∼ 2.15 and *n*_*e*_ ∼ 1.86 at *λ* = 594 nm)^42^. Additionally, MoS_2_ is known to possess extremely high optical anisotropy (*n*_*o*_ ∼ 4.7 and *n*_*e*_ ∼ 2.7 at *λ* = 750 nm) while being transparent from far-red to infrared^43^. Such a large birefringence reduces the propagation length required to convert the handedness of the CPL.

We experimentally demonstrate the generation of the optical vortex beam using an 8-µm-thick hBN crystal slab. The topological charge of the vortex is verified through an interferometer setup and numerical simulations. In addition, we experimentally obtain the spin-orbit conversion efficiencies of vortex generators and compare them with analytical predictions. The 8-µm-thick hBN crystal exhibits a conversion efficiency of 0.30 when a 594 nm laser is focused by an objective lens with 0.9 numerical aperture (NA). In addition, with the 26-µm-thick MoS_2_ crystal, we demonstrate a conversion efficiency of 0.46, which is close to the theoretical maximum value of 0.5. The thickness of the vortex generator can be further reduced to the sub-wavelength range. We observe the spin-orbit coupling in the 320 nm-thick MoS_2_ crystal flake, showing a conversion efficiency of 0.09. Finally, our simulations indicate that using a Bessel beam can further decrease the generator’s thickness and realize near-unity conversion efficiency. This work realizes the first application of spin-orbit coupling in vdW materials for generating optical vortex beams, opening an exciting avenue to create fabrication-free and versatile optical vortex generators feasible to both small- and large-scale experiments.

## Results

**Fig. 1:**
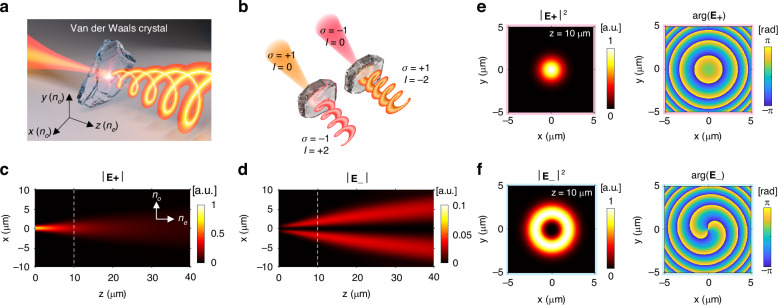
A schematic drawing of the optical vortex generation using a van der Waals crystals and simulations using a hexagonal boron nitride crystal. **a** A schematic illustration of the optical vortex generation due to spin-orbit coupling in a van der Waals (vdW) crystal. **b** (Left) left-handed circularly polarized (LCP) light (*σ* = +1), initially without orbital angular momentum (OAM) (*l* = 0), is incident onto a vdW crystal along its extraordinary axis, i.e., *z*-axis. As the beam propagates through the material, it is converted into a right-handed circularly polarized (RCP) beam (*σ* = −1) with an OAM mode of +2. (Right) When an RCP beam is incident, the vdW crystal converts it into an LCP beam with an OAM mode of −2 (*l* = −2). **c**, **d** A simulation of spin-orbit coupling in a hexagonal boron nitride (hBN) crystal. Amplitude distributions of the LCP and RCP fields, respectively, under the incidence of an LCP Gaussian beam with *λ* = 594 nm and a half-beam waist of 0.62 µm propagating along the extraordinary axis of the hBN crystal (*n*_*o*_ = 2.15, *n*_*e*_ = 1.86). |**E**_+_| and |**E**_−_| indicate the absolute amplitudes of the LCP and RCP electric fields, respectively, normalized by the maximum amplitude of the LCP field. The dashed lines at *z* = 10 µm indicate the section where the intensity and phase profiles in (**e**, **f**) are obtained. (**e**, **f**) Intensity (left) and phase profiles (right) of the LCP and RCP fields at *z* = 10 µm in the hBN crystal, respectively. The intensity profiles are normalized by their corresponding maximum intensities

Figure 1a schematically illustrates the creation of optical vortices via spin-orbit coupling in the hBN crystal. As shown in Fig. 1b, when a left-handed circularly polarized (LCP) beam (*σ* = +1) with zero topological charge (*l* = 0) is focused on the surface of a vdW crystal and propagates along the extraordinary axis, a portion of the incident beam is converted into a right-handed circularly polarized (RCP) beam (*σ* = −1) with OAM (*l* = +2) to conserve a total angular momentum. Conversely, if an RCP beam is incident, it generates an LCP beam with an OAM mode of *l* = −2. This conversion is confirmed by rigorous full-wave analysis^37,44,45^. The time-harmonic Maxwell’s equation of the complex electric field E is expressed as ${\nabla }^{2}{\bf{E}}-\nabla \left(\nabla \cdot {\bf{E}}\right)+{k}_{0}^{2}\varepsilon \cdot {\bf{E}}=0$, where *k*_0_ = 2*π/λ*. When the *z*-axis is the extraordinary axis of the uniaxial medium, a permittivity tensor *ε* can be written as follows:

1 $$\varepsilon =\left[\begin{array}{ccc}{n}_{o}^{2} & 0 & 0\\ 0 & {n}_{o}^{2} & 0\\ 0 & 0 & {n}_{e}^{2}\end{array}\right]$$

where *n*_*o*_ and *n*_*e*_ are ordinary and extraordinary refractive indices, respectively. Then, the electric field propagating in the *z*-direction can be derived by Fourier transforming the ordinary (o) and extraordinary (e) plane waves in the momentum space as

2 $${\bf{E}}\left({{\bf{r}}}_{\perp },z\right)={{\bf{E}}}_{o}\left({{\bf{r}}}_{\perp },z\right)+{{\bf{E}}}_{e}\left({{\bf{r}}}_{\perp },z\right)=\iint {d}^{2}{k}_{\perp }{e}^{i{{\bf{k}}}_{\perp }\cdot\, {{\bf{r}}}_{\perp }}\left[{\widetilde{u}}_{o}\left({{\bf{k}}}_{\perp }\right){e}^{i{k}_{{oz}}z}{\hat{{\bf{v}}}}_{o}\left({{\bf{k}}}_{\perp }\right)+{\widetilde{u}}_{e}\left({{\bf{k}}}_{\perp }\right){e}^{i{k}_{{ez}}z}{\hat{{\bf{v}}}}_{e}\left({{\bf{k}}}_{\perp }\right)\right]$$

where ${{\bf{k}}}_{\perp }={k}_{x}\hat{{\bf{x}}}+{k}_{y}\hat{{\bf{y}}}$ and ${{\bf{r}}}_{\perp }=x\hat{{\bf{x}}}+y\hat{{\bf{y}}}$ are the transverse wavevector and position vector, respectively, and ${\widetilde{u}}_{o,e}$ are the amplitudes of o- and e-plane wave modes. ${k}_{{oz}}={({k}_{0}^{2}{n}_{o}^{2}-{k}_{\perp }^{2})}^{1/2}$ and ${k}_{{ez}}={({k}_{0}^{2}{n}_{e}^{2}-{k}_{\perp }^{2})}^{1/2}{n}_{o}/{n}_{e}$ are the *z*-directional wavevectors of the o- and e-waves, respectively. ${\hat{{\bf{v}}}}_{o}=-\sin \phi \hat{{\bf{x}}}+\cos \phi \hat{{\bf{y}}}$ and ${\hat{{\bf{v}}}}_{e}=({k}_{{ez}}/{({k}_{{ez}}^{2}+{k}_{\perp }^{2})}^{1/2})({{\bf{k}}}_{\perp }/{k}_{\perp })-({k}_{\perp }/{({k}_{{ez}}^{2}+{k}_{\perp }^{2})}^{1/2})\hat{{\bf{z}}}$ are the unit vectors in the direction of the electric field of the o- and e- waves, and $\phi$ is the azimuthal angle of the plane wave’s Poynting vector. Under a paraxial condition, ${\hat{{\bf{v}}}}_{e}$ can be approximated as ${\hat{{\bf{v}}}}_{e}\simeq {{\bf{k}}}_{\perp }/{k}_{\perp }=\hat{{\bf{x}}}\cos \phi +\hat{{\bf{y}}}\sin \phi$, and the unit vectors of LCP ($+$) and RCP ($-$) waves follow ${\hat{{\bf{V}}}}_{\pm }\equiv (\hat{{\bf{x}}}\pm i\hat{{\bf{y}}})/\sqrt{2}\simeq {e}^{\pm i\phi }({\hat{{\bf{v}}}}_{e}\pm i{\hat{{\bf{v}}}}_{o})/\sqrt{2}$. By using this relation, we can derive the electric fields as a linear combination of LCP and RCP electric fields as

3 $${\bf{E}}\left({{\bf{r}}}_{\perp },z\right)={{\bf{E}}}_{+}\left({{\bf{r}}}_{\perp },z\right)+{{\bf{E}}}_{-}\left({{\bf{r}}}_{\perp },z\right)=\iint {d}^{2}{k}_{\perp }{e}^{i{{\bf{k}}}_{\perp }\cdot\, {{\bf{r}}}}\left[{\widetilde{U}}_{+}\left({{\bf{k}}}_{\perp },z\right){\widehat{\bf{V}}}_{+}+{\widetilde{U}}_{-}\left({{\bf{k}}}_{\perp },z\right){\widehat{\bf{V}}}_{-}\right]$$

Here, ${\widetilde{U}}_{\pm }\left({{\bf{k}}}_{\perp },z\right)$ are the amplitudes of the circularly polarized waves with topological charge of $l$ at propagation length z, which can be derived as

4 $$\left[\begin{array}{c}{\widetilde{U}}_{+}\left({{\bf{k}}}_{\perp },z\right)\\ {\widetilde{U}}_{-}\left({{\bf{k}}}_{\perp },z\right)\end{array}\right]=\left[\begin{array}{cc}({e}^{i{k}_{{ez}}z}+{e}^{i{k}_{{oz}}z}){e}^{{il}\phi }/2 & ({e}^{i{k}_{{ez}}z}-{e}^{i{k}_{{oz}}z}){e}^{i(l-2)\phi }/2\\ ({e}^{i{k}_{{ez}}z}-{e}^{i{k}_{{oz}}z}){e}^{i(l+2)\phi }/2 & ({e}^{i{k}_{{ez}}z}+{e}^{i{k}_{{oz}}z}){e}^{{il}\phi }/2\end{array}\right]\left[\begin{array}{c}{\widetilde{U}}_{+}\left({{\bf{k}}}_{\perp },0\right)\\ {\widetilde{U}}_{-}\left({{\bf{k}}}_{\perp },0\right)\end{array}\right]$$

The relation between the spin and orbital angular momentum can be obtained from Eq. 4. If an LCP beam with a topological charge *l* is incident, ${\widetilde{U}}_{+}\left({{\bf{k}}}_{\perp },0\right)={\widetilde{U}}_{+}\left({k}_{\perp },0\right)\exp ({il}\phi )$ and ${\widetilde{U}}_{-}\left({{\bf{k}}}_{\perp },0\right)=0$. Then, the amplitudes at propagation length *z* are ${\widetilde{U}}_{+}\left({{\bf{k}}}_{\perp },z\right)=[{(e}^{i{k}_{{ez}}z}+{e}^{i{k}_{{oz}}z}){e}^{{il}\phi }/2]{\widetilde{U}}_{+}\left({k}_{\perp },0\right)$ and ${\widetilde{U}}_{-}\left({{\bf{k}}}_{\perp },z\right)=[{(e}^{i{k}_{{ez}}z}-{e}^{i{k}_{{oz}}z}){e}^{i(l+2)\phi }/2]{\widetilde{U}}_{+}\left({k}_{\perp },0\right)$. This implies that the LCP beam with the OAM mode of *l* transfers its power to the RCP beam with the OAM mode of *l* + 2 as it propagates, following the total angular momentum conservation. In addition, the conversion efficiency of the wave with the transverse wavevector ${{\bf{k}}}_{\perp }$ is determined by ${|\exp (i{k}_{{ez}}z)-\exp (i{k}_{{oz}}z)|}^{2}$, implying that the phase difference between o- and e- waves due to the birefringence causes the spin-state conversion.

We conducted the cylindrical finite-difference time-domain (FDTD) simulation using open-source software MEEP^46^ to analyze the optical vortex generation in detail. Figure 1c, d show the electric field amplitude profiles of the LCP and RCP waves, respectively, illustrating the propagation of the LCP Gaussian beam in the hBN crystal (${n}_{o}=2.15$, ${n}_{e}=1.86$). The light source with a wavelength of 594 nm had a half-beam waist of 0.62 µm and propagates along the *z*-axis. Initially, the RCP intensity was zero at *z* = 0, as shown in Fig. 1d; it arose as the beam propagated along the crystal. Figure 1e, f show the intensity and phase profiles of the LCP and RCP waves, respectively. While the LCP wave maintained its Gaussian beam profile, the RCP wave displayed an annular intensity profile and a phase singularity at the center of the beam. In addition, the RCP wave displayed a 4*π* phase shift in the $\phi$-direction, indicating the OAM number of +2.

**Fig. 2:**
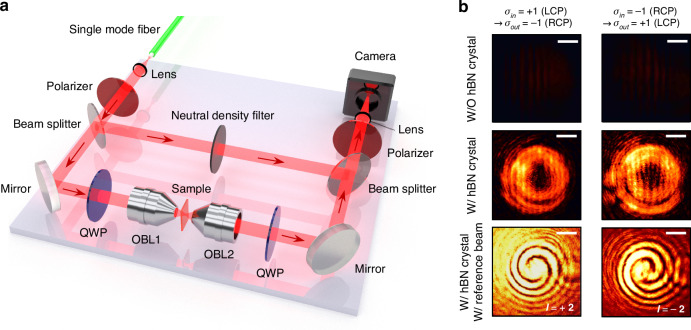
Demonstration of the optical vortex generation using the hBN crystal. **a** An experimental setup for vortex beam generation and characterization. An input beam toward the first objective lens (OBL1) is filtered to have left- or right-handed circular polarization. The focused beam from the OBL1 passes the sample and is collected by the second objective lens (OBL2). The LCP and RCP output beams from OBL2 are converted to two linearly polarized lights perpendicular to each other by a quarter wave plate (QWP). Finally, one of the linearly polarized lights passes through the linear polarizer (LP) and propagates to the camera as a signal beam. Two beam splitters and a neutral density filter are employed to make the signal beam interfere with a reference beam. **b** (Top and middle) Intensity profiles of the output beams when the input beam is focused on the bare glass substrate or on the hBN crystal on the substrate, respectively. (Bottom) Interference patterns of the output signal beams and the reference beam when the beam propagates the hBN crystal on the substrate. In the left column, the input beam toward the OBL1 is LCP, and only the RCP component of the output beam from the OBL2 passes the polarizer and propagates to the camera. In the right column, the input beam is RCP, and the LCP component of the output beam passes the polarizer. The numerical apertures (NAs) of the OBL1 and OBL2 are 0.4 and 0.42, respectively. The scale bar is 500 µm

We experimentally demonstrated the generation of the vortices in the hBN crystal. Figure 2a shows the interferometer setup we used for the vortex beam generation and characterization. We used a single-mode fiber and a lens to obtain a collimated transverse-electromagnetic (TEM) beam from a 594 nm laser. A linear polarizer (LP) and a quarter wave plate (QWP) created an LCP or RCP beam. The circularly polarized beam was subsequently focused by the first objective lens (OBL1), passed through the 8-µm-thick hBN crystal, and was collected by the second objective lens (OBL2). The QWP behind OBL2 converted LCP and RCP beams into two orthogonally polarized beams. Only the beam carrying the OAM was selected by the LP before a camera. Additionally, two beam splitters and a neutral density filter were used to obtain the interference pattern between the OAM-carrying beam and the reference beam. Figure 2b shows the intensity profiles captured by the camera. In the left column, the input beam to OBL1 was LCP, and only the RCP component of the output beam was collected and imaged by the camera using a QWP and an LP. Thus, we observed only the spin-converted beam from the input beam. In the right column, the input beam was RCP, and only the LCP component transmitted to the camera. When the beam propagates the glass-only sample (Fig. 2b, top), i.e., without hBN, the handedness of circular polarization was preserved; therefore, negligible intensities were measured in the camera. In contrast, when the focused beam transmits the hBN crystal placed on the substrate, annular intensity profiles were observed (middle row). From this result, we confirmed that the hBN crystal causes spin conversion of the incident beam.

Next, the topological charge number of the transmitted beam was verified using interferometry. Two spiral arms were observed when the reference beam, i.e., the fundamental transverse electromagnetic beam, interfered with the OAM beam. The number of arms and the direction of rotation indicate the topological charge and its sign, respectively. We observed two-armed spiral patterns rotating clockwise and counterclockwise under LCP and RCP inputs (bottom row), confirming the topological charge number of +2 and −2, respectively. These results were consistent with the simulation prediction shown in Fig. 1.

We also demonstrated the vortex generation using an MoS_2_ crystal. Using the same optical setup as the hBN crystals, but replacing the laser with 780 nm wavelength, we obtained output beam’s intensity profiles, as Fig. S1. The MoS_2_ generator exhibited the same tendency as the hBN crystal. The output beam displayed an annular intensity profile in both cases of the input beam’s polarization. Furthermore, the interference patterns of the output and reference beams exhibited two spiral arms, rotating clockwise and counterclockwise for LCP and RCP incidences, respectively.

The conversion efficiency *η* of the optical spin-orbit coupling in the vdW materials is defined by the ratio of the output vortex beam’s power to the total output power, showing the performance of the vortex generator. The conversion efficiency has been widely calculated using Gaussian-beam approximation^37,44,45^, which is described in the Supplementary material and Fig. S2. The approximated conversion efficiency is

5 $$\eta =\frac{1}{2}\left[1-\frac{1}{1+{(z/L)}^{2}}\right]$$

where $L={k}_{0}{n}_{o}{\omega }_{0}^{2}/({n}_{o}^{2}/{n}_{e}^{2}-1)$ is the anisotropic diffraction length^37^, and ${\omega }_{0}$ is half of the beam waist. Thus, the conversion efficiency depends on *z* and *L*, and its theoretical maximum value is 0.5.

**Fig. 3:**
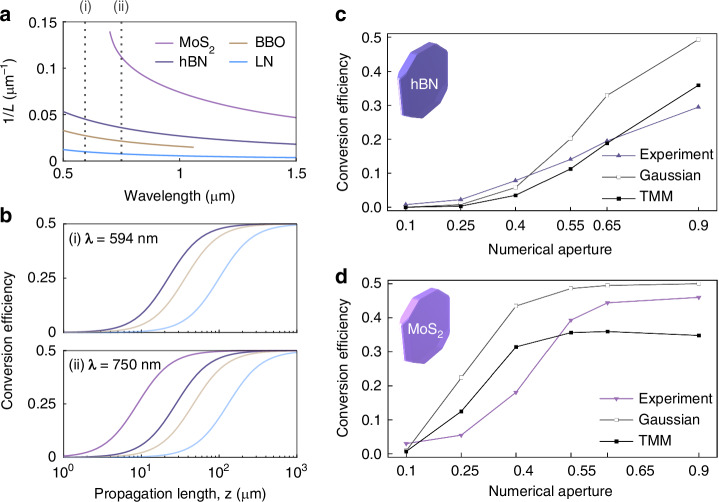
Predictions and measurements of the spin-orbit conversion efficiencies. **a** Inverses of anisotropic diffraction lengths (1*/L*) in the anisotropic materials (hBN, BBO, LN, and MoS_2_) by wavelength. The dashed lines at (i) *λ* = 594 nm and (ii) *λ* = 750 nm indicate wavelengths where the conversion efficiencies are predicted as shown in (**b**). Note that these wavelengths correspond to the wavelengths of the lasers used in the experiments. **b** Analytic predictions of the spin-orbit conversion efficiencies in the anisotropic materials by the propagation length at (i) *λ* = 594 nm and (ii) *λ* = 750 nm. **c**, **d** Calculations and measurements of the conversion efficiencies of a vortex generator with 8 µm-thick hBN and 26 µm-thick MoS_2_ crystals, respectively. The input beam’s wavelengths were 594 nm and 750 nm for the hBN and MoS_2_ crystals, respectively

Figure 3a shows the inverse of the anisotropic diffraction lengths, i.e., 1*/L*, in various uniaxial materials (hBN, LN, BBO, and MoS_2_) with respect to the wavelength. For the calculations using Eq. 5, we obtained the dispersion data of the uniaxial materials from the literature^39,40,42,43^ and set the half beam waist *w*_0_ to 0.62 µm. The propagation length to obtain the same conversion efficiency decreases with 1/L. Thus, MoS_2_ requires the lowest propagation length to achieve the same conversion efficiency, followed by hBN, due to their large optical anisotropy originating from the vdW structure. Figure 3b shows calculated conversion efficiencies depending on the propagation length. In addition, two wavelengths, 594 nm for hBN and 750 nm for MoS_2_, are selected for the experiments to ensure high conversion efficiencies and low absorptions.

The Gaussian beam approximation provides insights into the conversion efficiency. It has shown significant agreement with the experimental results of the spin-orbit conversion in thick uniaxial crystals in previous studies^14,15^. However, the experiments with the high-NA lenses and thin crystals cannot be accurately demonstrated because they use paraxial approximation and do not consider the reflections at the interfaces of the crystals. Thus, we suggest another calculation method using the transfer-matrix method (TMM). We assume the input and output objective lenses, OBL1 and OBL2, are perfect Fourier transformers. We also assume that the vdW crystals are dielectric slabs with finite thicknesses, whose interfaces are orthogonal with the extraordinary axis. The detailed calculation methods are provided in the Supplementary material.

Figure 3c displays the experimental results (triangle dots) and calculations (square dots) of the conversion efficiencies for a vortex generator with varying NAs of OBL1, where the 8-µm-thick hBN crystal (Fig. S3a, b) is used. The measurement setup is shown in Fig. S4, and half of the beam waist ${\omega }_{0}$ was set to 0.42*λ/*NA by Gaussian beam approximation^47^. In addition, we used the crystal thickness as the value of the propagation length *z* for the Gaussian beam approximation. Detailed information about the vdW samples and measurement methods is provided in the Supplementary material. In a low NA region (NA ≤ 0.4), the experimental results almost matched both calculations. However, the experimental results were lower than the Gaussian beam approximation in the high NA region (NA ≥ 0.55). The difference between the Gaussian beam approximation and the experimental result may be due to the paraxial approximation and the polarization-dependent transmissions (Fig. S6). On the other hand, the TMM-based calculations were lower than the experiments, which can be attributed to errors in the assumption of the input beam’s amplitudes.

Figure 3d shows the conversion efficiencies of the vortex generator using the 26 µm-thick MoS_2_ crystal (Figs. S3c–e) depending on the NA of OBL1. We used a 750 nm laser for the experiment and used its wavelength for the calculations. Due to the high optical anisotropy of the MoS_2_ crystal, the conversion efficiency quickly saturated and reached near 0.5 as the NA increased, compared to that of the hBN crystal. We report the maximum conversion efficiency of 0.46 using NA of 0.9 in this work. Next, the thickness is reduced to below µm to realize spin-orbit coupling in a sub-wavelength scale. We achieved the experimental conversion efficiency of 0.09 using 320 nm-thick MoS_2_ and 0.9 NA objective lens, where the Gaussian beam approximation is 0.16 (Fig. S5).

A use of a circularly polarized Bessel beam can overcome the Gaussian beam’s maximum conversion efficiency limit of 0.5, enabling the near-unity conversion efficiency^45,48,49^. The Bessel beam is a beam whose amplitude is a Bessel function of the first kind, and it can be described as a superposition of plane waves having a single value of the transverse wavevector *k*_⊥_ and cylindrically symmetric amplitudes^45,50^. An ideal LCP Bessel beam can be expressed as ${\bf{E}}\left({{\bf{r}}}_{\perp },0\right)={A}_{0}{J}_{0}({k}_{t}{r}_{\perp }){\hat{{\bf{V}}}}_{+}$ in real space and ${\widetilde{U}}_{+}\left({{\bf{k}}}_{\perp },0\right)={\widetilde{U}}_{0}\delta ({k}_{\perp }-{k}_{t})$ in the Fourier space, where ${A}_{0}$ and ${\widetilde{U}}_{0}$ are arbitral constant amplitudes, and *k*_*t*_ is the constant transverse wavevector. In addition, *J*_*n*_ is the first kind of Bessel function with an order of *n*, and *δ* is a Dirac delta function. As described in Eq. 4, the spin-orbit conversion efficiency at the transverse wavevector **k**_⊥_ is determined by its value *k*_⊥_ and can reach unity. Moreover, we can achieve both near-unity conversion efficiency and transmission by controlling the crystal thickness and transverse wavevector, as shown in Fig. S6b.

**Fig. 4:**
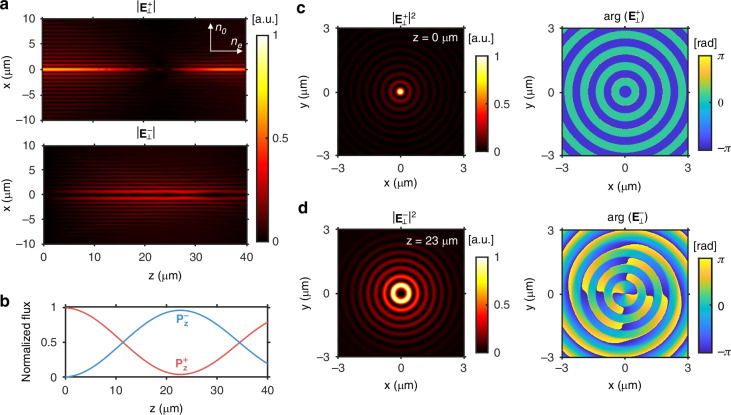
Spin-orbit coupling of a Bessel beam in an hBN crystal. **a** Amplitude distribution of a circularly polarized Bessel beam propagating in the hBN crystal along the extraordinary axis when *λ* = 594 nm. The $|{{\bf{E}}}_{\perp }^{+}|$ and $|{{\bf{E}}}_{\perp }^{-}|$ indicate the absolute amplitudes of the LCP and RCP electric fields, respectively, normalized by the maximum amplitude of the LCP wave. **b** *z*-directional powers of the LCP (${P}_{z}^{+}$, red line) and RCP (${P}_{z}^{-}$, blue line) waves. **c** Intensity and phase profiles of the LCP wave at *z* = 0 *µ*m (**d**) and of the RCP wave at *z* = 23 µm. The intensity profiles are normalized by their respective maximum intensities

Figure 4a shows the amplitude profiles of the LCP and RCP fields of the Bessel beam propagating along the extraordinary axis of the hBN crystal at *λ* = 594 nm. The LCP Bessel beam was generated at *z* = 0 µm with a diameter of 10 µm and a transverse wavevector *k*_*t*_ of 0.4 *k*_0_. As *z* increased from 0 to 23 µm, the LCP field almost disappeared, and the RCP field intensity rose. Figure 4b shows the normalized *z*-directional powers of the LCP and RCP waves. Those powers oscillated sinusoidally, and the RCP power showed the highest value of 0.96 at *z* = 23 µm. Figure 4c and d show the intensity and phase profiles of the LCP and RCP components, respectively, revealing an RCP Bessel beam with the OAM mode of +2 at *z* = 23 µm. The conversion efficiency of the Bessel beam can be analytically derived from Eq. 4 as

6 $${\eta }_{B}=\frac{1}{4}{\left|\exp (i{k}_{{ez}}z)-\exp (i{k}_{{oz}}z)\right|}^{2}={\sin }^{2}\left(\frac{{k}_{{oz}}-{k}_{{ez}}}{2}z\right)$$

In the case of the hBN crystal, *η*_*B*_ approaches unity at *z* = 23 µm as the simulation shows. We can further reduce the propagation length for the near-unity spin-orbit conversion efficiency by increasing the transverse wavevector *k*_*t*_ or by using a medium with a larger birefringence. For instance, we can reduce the minimum length for the near-unity conversion efficiency by replacing the hBN crystal with the MoS_2_ crystal. Figure S7 shows the Bessel beam simulation using the MoS_2_ crystal. The simulated minimum length for the near-unity conversion efficiency was 10.3 µm, closely matching 10.7 µm calculated from Eq. 6.

## Discussion

Table 1 presents a comparison of microscale vortex generators, categorized into three types: metasurface, waveguide, and spin-orbit coupling. The metasurfaces and waveguide types exhibit high efficiencies and diverse functionalities, such as multi-OAM generations^30^ or fiber-to-waveguide couplings^11^. However, they require precise nanofabrications. On the contrary, the spin-orbit coupling type can be realized without the need for nanofabrication. In this type, self-assembled liquid crystal dimples^23^ have demonstrated high efficiency (∼ 0.5) but were limited to the micrometer scale. Meanwhile, epsilon-near-zero slabs^48^ have achieved sub-wavelength thickness but suffered from limited bandwidth and efficiency. In contrast, MoS_2_ crystals exhibit a combination of broad bandwidth spanning several micrometers, sub-wavelength thickness, and applicable efficiency. These characteristics underscore the potential use of vdW material-based optical vortex generators for integrated photonics.

**Table 1:** Comparison of microscale optical vortex generators

Type	Representative works [ref]	Nano- fabrication	Thickness (nm)	Wavelength (nm)	Bandwidth* (nm)	Efficiency*
Metasurface	Geometric metasurfaces^51^	O	600	530	∼200	∼1
	Resonant metasurface^30^	O	800	532	N/A	∼1
Waveguide	Ring microcomb^31^	O	380	1550	N/A	N/A
	Free-form emitter^11^	O	850	1570	∼100	∼0.3
	Mode converter^52^	O	500	1550	N/A	0.05
Spin-orbit	Liquid crystal dimples^23^	X	8 × 10^3^	632	N/A	∼0.5
coupling	Epsilon-near-zero slab^48^	X	∼100	1240	∼100	∼0.06
	MoS_2_ crystal (This work)	X	320 /2.6 × 10^4^	750	>10^3^	0.09 / 0.46

^*^The bandwidth is defined as the wavelength range where the efficiency remains greater than half of its maximum value

^*^The efficiency of the metasurface and waveguide types is determined by following the methodology outlined in the corresponding references. Furthermore, the efficiency of the spin-orbit coupling type corresponds to the spin-orbit conversion efficiency

In conclusion, we present the generation of the optical vortex beam by leveraging the spin-orbit coupling in vdW materials, eliminating the need for fabrication processes. By utilizing the hBN crystal, we produced the annular beam profile with the topological charge of ±2 through the conversion of circular polarization. The conversion efficiencies of the vortex generators were measured, revealing that the 8 µm-thick hBN crystal and the 26 µm-thick MoS_2_ crystal achieved maximum conversion efficiencies of 0.30 and 0.46, respectively. Furthermore, we demonstrated spin-orbit coupling on the sub-wavelength scale, achieving the conversion efficiency of 0.09 using the 320 nm-thick MoS_2_ crystal flake. In addition, our numerical simulations further suggest that near-unity conversion efficiency can be realized in vdW vortex generators by employing Bessel beams. These ultra-compact, fabrication-free vortex beam generators represent a potential in nanophotonics, particularly in applications involving orbital angular momentum.

## Materials and methods

### Simulation method

We utilized cylindrically symmetric FDTD simulations, whose mesh sizes were set to 10 nm along both *r*_⊥_- and z-directions. The boundaries of the simulation space were surrounded by 1-μm-thick perfect matching layers to absorb outgoing waves. In the Gaussian beam simulation, the radius and length of the simulation space were 11 and 21.1 μm, respectively, and the source’s radius was 10 μm. In the Bessel beam simulation, the radius of the simulation space and the source were increased to 21 and 20 μm, respectively.

### Sample preparation and characterization

The hBN and MoS_2_ crystals were purchased from 2D Semiconductors and placed on 0.15 mm-thick glass substrates. Then, their optical microscope (OM) images were taken as shown in Fig. S3a, c. The thicknesses of both crystals were measured using a Bruker Contour GT-I profilometer with a green light source, as shown in Fig. S3b, d. The sub-wavelength thick MoS_2_ crystal flake was obtained from a MoS_2_ crystal by mechanical exfoliation using a thermal release tape and was transferred on the 0.15-mm-thick glass substrate. The substrate was prepared after cleaning with acetone, distilled water, and isopropyl alcohol. The thickness of the MoS_2_ flake was measured as depicted in Fig. S5a using an atomic force microscope (AFM) of Asylum Research Cypher.

## Supplementary information

Supplementary Material

## Data Availability

The data that support the findings of this study are available from the corresponding author upon reasonable request.
