# Supplementary material for: Spin-orbit coupling in van der Waals materials for optical vortex generation
Source: Light Sci Appl. 2025 Aug 18;14:277. doi: 10.1038/s41377-025-01926-7 (PMC12358514; doi:10.1038/s41377-025-01926-7)
Supplement: Supplementary file 1 — Supplementary Material [file 41377_2025_1926_MOESM1_ESM.pdf]

# Supplementary Information for Spin-Orbit Coupling in van der Waals Materials for Optical Vortex Generation

Jaegang Jo[1,†], Sujeong Byun[2,†], Munseong Bae[1], Jianwei Wang[3], Haejun Chung[1,a,*], and Sejeong Kim[2,b,*]

[1]Department of Electronic Engineering, Hanyang University, Seoul, 04763, Republic of Korea

[2]Department of Electrical and Electronic Engineering, Faculty of Engineering and Information Technology, University of Melbourne, Melbourne 3000, Australia

[3]State Key Laboratory for Mesoscopic Physics and Collaborative Innovation Center of Quantum Matter, School of Physics, Peking University, Beijing, 100871, China

[†]These authors contributed equally to this work.

[*]These authors are corresponding authors.

June 29, 2025

[a] haejun@hanyang.ac.kr [b] sejeong.kim@unimelb.edu.au

# Contents

**1 Calculation methods** **2**

1.1 Finite-difference time-domain (FDTD) simulation methods . . . 2

1.2 Conversion efficiency by Gaussian-beam approximation . . . 2

1.3 Conversion efficiency by transfer-matrix method . . . 5

**2 Preparation and characterization of vdW crystal samples** **7**

**3 Optical setup** **8**

3.1 Conversion efficiency measurements . . . 8

3.2 Light source equipments . . . 9

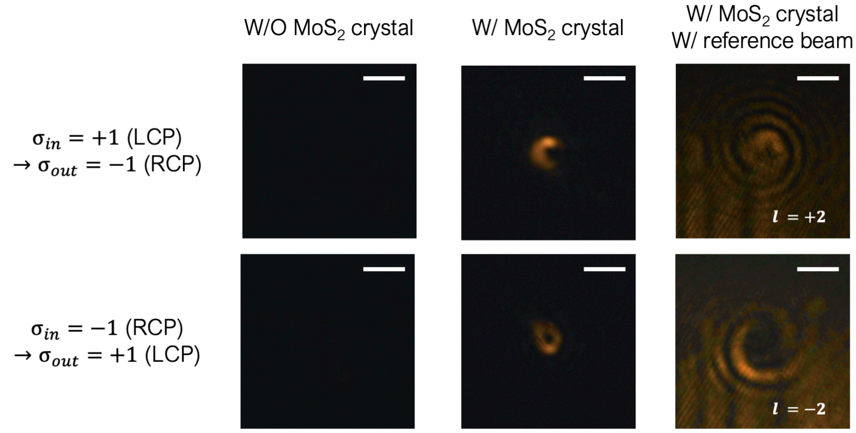


Figure S1: (Left and center) Intensity profiles of the output beams when the input beam is focused on the glass substrate or on the $MoS_2$ crystal on the glass substrate, respectively. (Right) Interference patterns created by the reference and output beams. In the top row, the input beam is LCP, and the output beam is filtered as RCP. In the bottom row, the input and output beam's polarizations are opposite to the top row. The wavelength of the input beam is 780 nm, and the thickness of the $MoS_2$ crystal is approximately 16 µm. The scale bar is 500 µm.

# 1 Calculation methods

## 1.1 Finite-difference time-domain (FDTD) simulation methods

For the simulations involving Gaussian beam incidence, we utilized a cylindrical symmetry with a simulation domain of 22.1 µm in length and 11 µm in radius. The grid spacing was set to 10 nm along both the $z$-axis and the radial coordinate $r_\perp$ of the cylindrical coordinate system. The boundaries of the simulation space were surrounded by 1-µm-thick perfect matching layers (PMLs) to absorb all outgoing waves. A source plane was positioned 20 nm to the right from the left boundary ($-z$). The entire simulation space was filled with the hBN crystal having refractive indices of $n_o = 2.15$ and $n_e = 1.86$. For the Bessel beam simulation with the hBN crystal, the simulation space's radius and length were increased to 21 µm and 42.2 µm, respectively. In addition, we applied 10 nm grid spacing, 21 µm radius, and 22.2 µm length for the Bessel beam simulation with the $MoS_2$ crystal. The refractive indices of the $MoS_2$ were $n_o = 4.7$ and $n_e = 2.7$.

## 1.2 Conversion efficiency by Gaussian-beam approximation

Let an LCP Gaussian beam have a beam waist $w_0$ and waist plane at $z = 0$. The electric field at $z = 0$ is

$$\mathbf{E}_+(\mathbf{r}_\perp, 0) = E_0 \exp\left(-r_\perp^2/w_0^2\right) \hat{\mathbf{V}}_+, \tag{1}$$

where $\mathbf{r}_\perp = x\hat{\mathbf{x}} + y\hat{\mathbf{y}}$, and $w_0$ is the half width at beam waist. The amplitude at the momentum space can be obtained from the Fourier transform as

$$\tilde{U}_+(\mathbf{k}_\perp, 0) = \tilde{U}_0 \exp\left[-(w_0 k_\perp)^2/4\right] \tag{2}$$

Furthermore, from Eq. 4, the amplitudes of the LCP and RCP components with $z$ are

$$\tilde{U}_+(\mathbf{k}_\perp, z) = \left[\left(e^{ik_{ez}z} + e^{ik_{oz}z}\right)/2\right]\tilde{U}_+(k_\perp, 0) \tag{3}$$

$$\tilde{U}_-(\mathbf{k}_\perp, z) = \left[\left(e^{ik_{ez}z} - e^{ik_{oz}z}\right)/2\right]\tilde{U}_+(k_\perp, 0)\exp(i2\phi). \tag{4}$$

The conversion efficiency $\eta$ is defined by

$$\eta(z) \equiv \frac{\iint \left|\tilde{U}_-(\mathbf{k}_\perp, z)\right|^2 dk_\perp^2}{\iint \left|\tilde{U}_+(\mathbf{k}_\perp, 0)\right|^2 dk_\perp^2}. \tag{5}$$

The denominator is

$$\iint \left|\tilde{U}_-(\mathbf{k}_\perp, z)\right|^2 dk_\perp^2 = \iint \left|\sin\left[(k_{ez} - k_{oz})\, z/2\right]\tilde{U}_+(k_\perp, 0)\right|^2 dk_\perp^2 \tag{6}$$

$$= \iint \frac{1}{2}\left\{1 - \cos\left[(k_{ez} - k_{oz})\, z\right]\right\}\left|\tilde{U}_+(k_\perp, 0)\right|^2 dk_\perp^2. \tag{7}$$

From paraxial approximation, $k_{oz} = (k_0^2 n_o^2 - k_\perp^2)^{1/2} \simeq k_0 n_o - k_\perp^2/2k_0 n_o$ and $k_{ez} = (k_0^2 n_e^2 - k_\perp^2)^{1/2} n_o/n_e \simeq k_0 n_o - n_o k_\perp^2/2k_0 n_e^2$. Thus, the denominator of the conversion efficiency is

$$\iint \left|\tilde{U}_-(\mathbf{k}_\perp, z)\right|^2 dk_\perp^2 = \iint \frac{1}{2}\left\{1 - \cos\left[\left(-\frac{k_\perp^2}{2k_0 n_o} + \frac{n_o k_\perp^2}{2k_0 n_e^2}\right) z\right]\right\}\left|\tilde{U}_+(k_\perp, 0)\right|^2 dk_\perp^2 \tag{8}$$

$$= \iint \frac{1}{2}\left[1 - \cos\left(\frac{k_\perp^2 w_0^2 z}{2L}\right)\right]\left|\tilde{U}_+(k_\perp, 0)\right|^2 dk_\perp^2, \tag{9}$$

where $L = k_0 n_o w_0^2/(n_o^2/n_e^2 - 1)$ is the anisotropic diffraction length. Consequently, the conversion efficiency depends on $z$ as

$$\eta = \frac{1}{2}\left[1 - \frac{1}{1 + (z/L)^2}\right]. \tag{10}$$

From Eq. 9, we can find that the intensity of the spin-converted beam is the integral of the multiplication of the conversion efficiency function $\tilde{\eta}(k_\perp, z) = \left[1 - \cos\left(k_\perp^2 w_0^2 z/2L\right)\right]/2$ and the original beam's intensity $|\tilde{U}_+(k_\perp, 0)|^2$. As shown in Fig. S2, the conversion efficiency function is shaped as numerous rings with the same center at $k_\perp = 0$. The spacings between rings decrease with z, therefore, we can estimate that the conversion efficiency converges to 1/2 as $z$ increases.

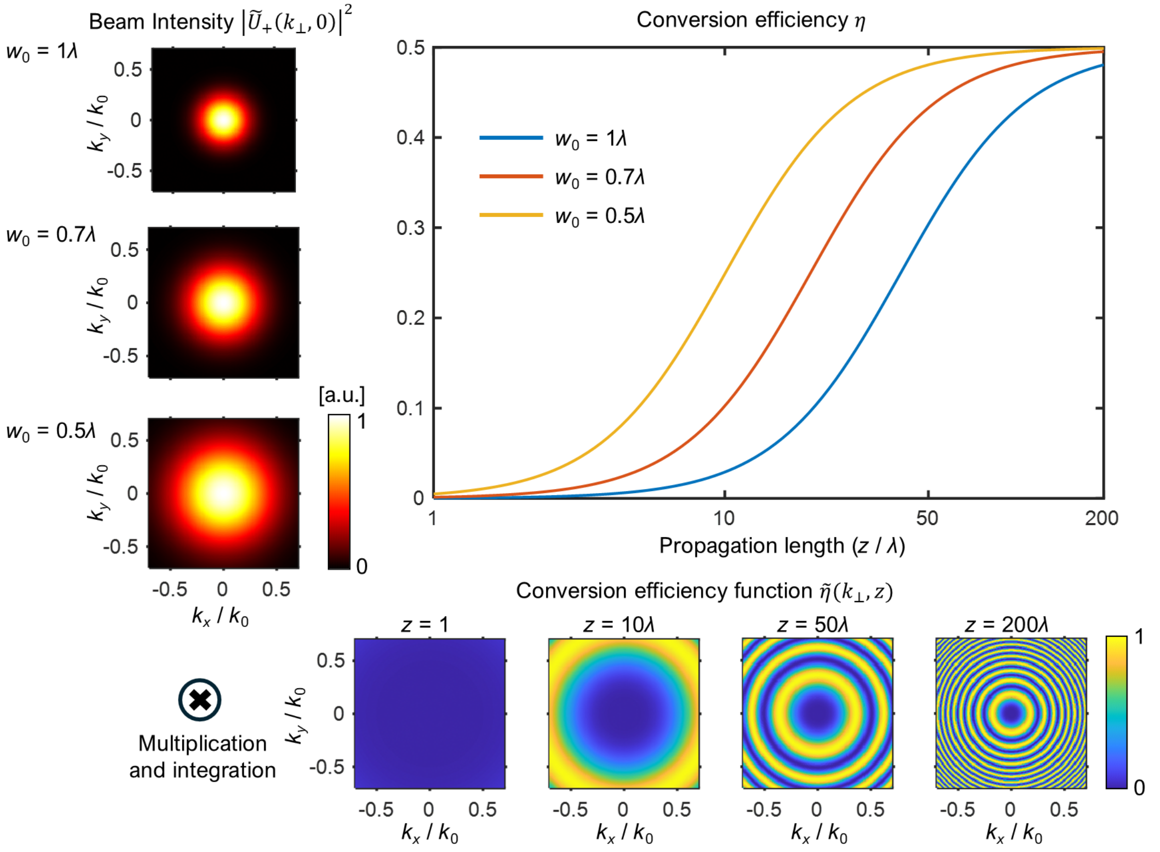


Figure S2: Conversion efficiency of circularly polarized Gaussian beam in hBN crystal. $n_o = 2.15$ and $n_e = 1.86$ are obtained from refractive indices of the hBN crystal at $\lambda = 594$ nm and are used for the calculation. Left figures show the Gaussian beam's intensities $|\tilde{U}_+(k_\perp, 0)|^2$ with different beam waists $w_0$, and bottom figures indicate conversion efficiency functions $\tilde{\eta}(k_\perp, z)$ depending on $z$ . A graph at the center shows the conversion efficiencies calculated by the integrations of the multiplications of $|\tilde{U}_+(k_\perp, 0)|^2$ and $\tilde{\eta}(k_\perp, z)$.

## 1.3 Conversion efficiency by transfer-matrix method

Let an uniaxial crystal slab extend infinitely in the $xy$-direction, with an extraordinary axis parallel to the $z$-axis and boundary interfaces at $z = 0$ and $z = d$. The electric field can be expressed as a linear combination of p- and s-polarized fields as

$$\mathbf{E}(\mathbf{r}_\perp, z) = \begin{cases} \mathbf{E}_s^+(\mathbf{r}_\perp, z) + \mathbf{E}_s^-(\mathbf{r}_\perp, z) + \mathbf{E}_p^+(\mathbf{r}_\perp, z) + \mathbf{E}_p^-(\mathbf{r}_\perp, z) & z > 0 \quad \text{or} \quad z > d \\ \mathbf{E}_o^+(\mathbf{r}_\perp, z) + \mathbf{E}_o^-(\mathbf{r}_\perp, z) + \mathbf{E}_e^+(\mathbf{r}_\perp, z) + \mathbf{E}_e^-(\mathbf{r}_\perp, z) & 0 < z < d \end{cases} \tag{11}$$

where $\mathbf{E}_{s/p}^\pm(\mathbf{r}_\perp, z)$ is s- or p-polarized wave propagating to $+z$- or $-z$-direction, and $\mathbf{E}_{o/e}^\pm(\mathbf{r}_\perp, z)$ is o- or e-wave propagating to $+z$- or $-z$-direction. The fields can be expressed in Fourier space as

$$\mathbf{E}_{s/p}^\pm(\mathbf{r}_\perp, z) = \iint d^2k_\perp e^{i\mathbf{k}_\perp\cdot\mathbf{r}_\perp} \tilde{u}_{s/p}^\pm(\mathbf{k}_\perp, z)\hat{\mathbf{v}}_{s/p}^\pm(\mathbf{k}_\perp) \tag{12}$$

$$\mathbf{E}_{o/e}^\pm(\mathbf{r}_\perp, z) = \iint d^2k_\perp e^{i\mathbf{k}_\perp\cdot\mathbf{r}_\perp} \tilde{u}_{o/e}^\pm(\mathbf{k}_\perp, z)\hat{\mathbf{v}}_{o/e}^\pm(\mathbf{k}_\perp). \tag{13}$$

Here, $k_z = (k_0^2 - k_\perp^2)^{1/2}$, and $\tilde{u}_{o/e}^\pm$ and $\tilde{u}_{s/p}^\pm$ are amplitudes of o/e- and s/p-waves propagating to $\pm z$-direction, respectively. Furthermore, the unit vectors of the electric fields, $\hat{\mathbf{v}}_{s/p}^\pm(\mathbf{k}_\perp)$ and $\hat{\mathbf{v}}_{o/e}^\pm(\mathbf{k}_\perp)$ are

$$\hat{\mathbf{v}}_s^\pm(\mathbf{k}_\perp) = \hat{\mathbf{v}}_o^\pm(\mathbf{k}_\perp) = -\sin\phi\hat{\mathbf{x}} + \cos\phi\hat{\mathbf{y}} \tag{14}$$

$$\hat{\mathbf{v}}_p^\pm(\mathbf{k}_\perp) = (k_z/k_0)\,(\mathbf{k}_\perp/k_\perp) \mp (k_\perp/k_0)\,\hat{\mathbf{z}} \tag{15}$$

$$\hat{\mathbf{v}}_e^\pm(\mathbf{k}_\perp) = \left(k_{ez}/(k_{ez}^2 + k_\perp^2)^{1/2}\right)(\mathbf{k}_\perp/k_\perp) \mp \left(k_\perp/(k_{ez}^2 + k_\perp^2)^{1/2}\right)\hat{\mathbf{z}}. \tag{16}$$

By applying boundary conditions at $z = 0$ and $z = d$, we can obtain a transfer-matrix equation as

$$\begin{bmatrix} \tilde{u}_s^+(\mathbf{k}_\perp, 0) \\ \tilde{u}_s^-(\mathbf{k}_\perp, 0) \\ \tilde{u}_p^+(\mathbf{k}_\perp, 0) \\ \tilde{u}_p^-(\mathbf{k}_\perp, 0) \end{bmatrix} = \mathbf{M} \begin{bmatrix} \tilde{u}_s^+(\mathbf{k}_\perp, d) \\ \tilde{u}_s^-(\mathbf{k}_\perp, d) \\ \tilde{u}_p^+(\mathbf{k}_\perp, d) \\ \tilde{u}_p^-(\mathbf{k}_\perp, d) \end{bmatrix} = \mathbf{I}\mathbf{P}^{-1}\mathbf{I}^{-1} \begin{bmatrix} \tilde{u}_s^+(\mathbf{k}_\perp, d) \\ \tilde{u}_s^-(\mathbf{k}_\perp, d) \\ \tilde{u}_p^+(\mathbf{k}_\perp, d) \\ \tilde{u}_p^-(\mathbf{k}_\perp, d) \end{bmatrix}. \tag{17}$$

Here, $\mathbf{P}$ and $\mathbf{I}$ are phase-delay and interface matrices as

$$\mathbf{P} = \begin{bmatrix} e^{ik_{oz}d} & 0 & 0 & 0 \\ 0 & e^{-ik_{oz}d} & 0 & 0 \\ 0 & 0 & e^{ik_{ez}d} & 0 \\ 0 & 0 & 0 & e^{-ik_{ez}d} \end{bmatrix}, \tag{18}$$

$$\mathbf{I} = \frac{1}{2}\begin{bmatrix} 1+k_{oz}/k_z & 1-k_{oz}/k_z & 0 & 0 \\ 1-k_{oz}/k_z & 1+k_{oz}/k_z & 0 & 0 \\ 0 & 0 & 1+k_z(k_{ez}^2+k_\perp^2)/k_{ez}k_0^2 & 1-k_z(k_{ez}^2+k_\perp^2)/k_{ez}k_0^2 \\ 0 & 0 & 1-k_z(k_{ez}^2+k_\perp^2)/k_{ez}k_0^2 & 1+k_z(k_{ez}^2+k_\perp^2)/k_{ez}k_0^2 \end{bmatrix}. \quad (19)$$

When a beam is incident from -z direction, $\tilde{u}_s^-(\mathbf{k}_\perp, d) = \tilde{u}_p^-(\mathbf{k}_\perp, d) = 0$. Thus, the transmitting amplitudes are $\tilde{u}_s^+(\mathbf{k}_\perp, d) = \tilde{u}_s^+(\mathbf{k}_\perp, 0)/\mathbf{M}_{11}(k_\perp)$ and $\tilde{u}_p^+(\mathbf{k}_\perp, d) = \tilde{u}_p^+(\mathbf{k}_\perp, 0)/M_{33}(\mathbf{k}_\perp)$, where $M_{11}(\mathbf{k}_\perp)$ and $M_{33}(\mathbf{k}_\perp)$ are components of $\mathbf{M}$.

We assume that the incident beam toward the sample is an LCP beam with a Gaussian intensity distribution. Then, by using the amplitudes in Eq. 2, we can write the field amplitudes at $z = 0$ as

$$\tilde{u}_s^+(\mathbf{k}_\perp, 0) = \frac{i}{\sqrt{2}} e^{i\phi} \tilde{u}_0 \exp\left[-(w_0 k_\perp)^2/4\right] \Pi(k_\perp - k_0\mathrm{NA}) \quad (20)$$

$$\tilde{u}_p^+(\mathbf{k}_\perp, 0) = \frac{1}{\sqrt{2}} e^{i\phi} \tilde{u}_0 \exp\left[-(w_0 k_\perp)^2/4\right] \Pi(k_\perp - k_0\mathrm{NA}). \quad (21)$$

Here, NA is a numerical aperture of the objective lens, and $w_0 = 0.42\lambda/\mathrm{NA}$ is a half of beam waist. Furthermore, $\tilde{u}_0$ is an arbitral constant amplitude, and $\Pi(k_\perp - k_0\mathrm{NA})$ is a step function, which is 1 at $0 \le k_\perp < k_0\mathrm{NA}$ and 0 at $k_\perp \ge k_0\mathrm{NA}$. The transmitted spin-converted power $P_{\mathrm{SC}}$ is

$$P_{\mathrm{SC}} = \iint |(i/\sqrt{2})\tilde{u}_s^+(\mathbf{k}_\perp, d) + (1/\sqrt{2})\tilde{u}_p^+(\mathbf{k}_\perp, d)|^2 dk_\perp^2 \quad (22)$$

$$= \iint \frac{1}{4}|1/M_{11}(\mathbf{k}_\perp) - 1/M_{33}(\mathbf{k}_\perp)|^2 \tilde{u}_0^2 \exp\left[-(w_0 k_\perp)^2/2\right] \Pi(k_\perp - k_0\mathrm{NA}) dk_\perp^2. \quad (23)$$

Likewise, the total transmitted power $P_{\mathrm{Total}}$ is

$$P_{\mathrm{Total}} = \iint |(i/\sqrt{2})\tilde{u}_s^+(\mathbf{k}_\perp, d)|^2 + |(1/\sqrt{2})\tilde{u}_p^+(\mathbf{k}_\perp, d)|^2 dk_\perp^2 \quad (24)$$

$$= \iint \frac{1}{2}\left(|1/M_{11}(\mathbf{k}_\perp)|^2 - |1/M_{33}(\mathbf{k}_\perp)|^2\right) \tilde{u}_0^2 \exp\left[-(w_0 k_\perp)^2/2\right] \Pi(k_\perp - k_0\mathrm{NA}) dk_\perp^2. \quad (25)$$

In conclusion, the spin-orbit conversion efficiency $\eta$ is $P_{\mathrm{SC}}/P_{\mathrm{Total}}$. In addition, $T_{\mathrm{SC}}(\mathbf{k}_\perp) = (|1/M_{11}(\mathbf{k}_\perp) - 1/M_{33}(\mathbf{k}_\perp)|^2/4$ and $T_{\mathrm{Total}}(\mathbf{k}_\perp) = \left(|1/M_{11}(\mathbf{k}_\perp)|^2 - |1/M_{33}(\mathbf{k}_\perp)|^2\right)/2$ are spin-converted and total transmission of a plane wave with a transverse wave-vector $\mathbf{k}_\perp$, respectively.

# 2 Preparation and characterization of vdW crystal samples

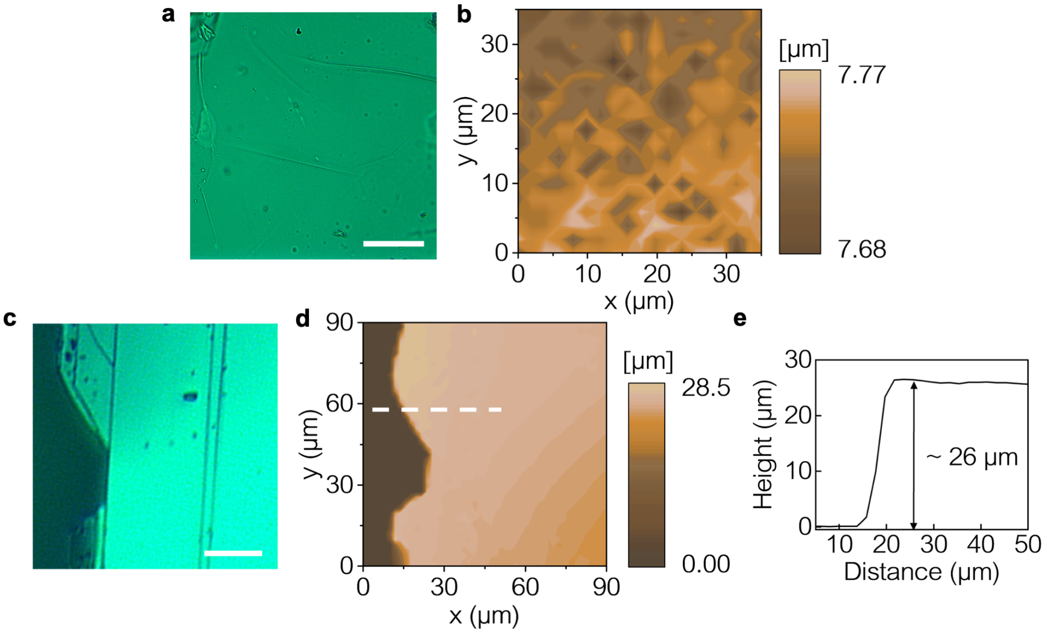


Figure S3: Optical microscope (OM) images and thickness profiles of the hBN and $MoS_2$ crystals. (a) An OM image of the hBN crystal where the conversion efficiency is measured. The scale bar is 20 μm. (b) A thickness profile at the corresponding area. (c) An OM image of the $MoS_2$ crystal where the conversion efficiency is measured. The scale bar is 20 μm. (d) A thickness profile at the corresponding area. (e) An one-dimensional thickness profile at the white dashed line indicated in (d). Note that the profilometer can obtain accurate thickness even in areas without a crystal edge because it uses optical interference in the crystals.

Fig. S3a and b show an OM image and a two-dimensional thickness profile of the hBN crystal in the area where the vortex beam's intensity profile and the conversion efficiency are measured. The overall thickness of the hBN crystal is approximately 8 μm. Fig. S3c and d also show the OM image and the thickness profiles of the $MoS_2$ crystal where conversion efficiency is measured. Furthermore, Fig. S3e shows the one-dimensional profile of the dashed line in Fig. S3d. The $MoS_2$ crystal is about 26-μm-thick.

# 3 Optical setup

## 3.1 Conversion efficiency measurements

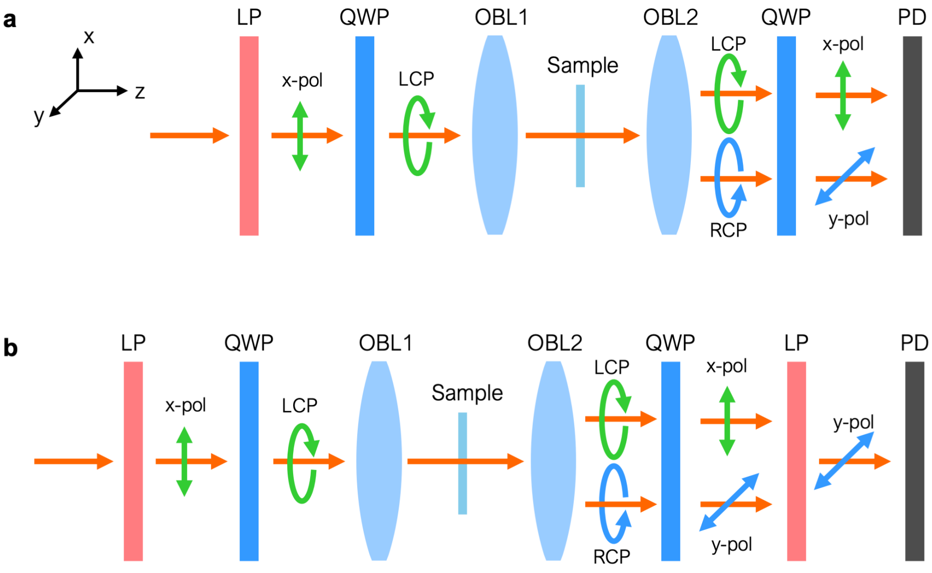


Figure S4: Optical setups for conversion efficiency measurements. (a) An optical setup for total output power ($P_{\mathrm{Tot}}$) measurement. The photodiode (PD) detects the power of both left- and right-handed circularly polarized (LCP and RCP) beams from the second objective lens (OBL2), while the input beam into the first objective lens (OBL1) is LCP. (b) An optical setup for spin-converted output power ($P_{\mathrm{SC}}$) measurement. The setup is the same as the total output power measurement setup except for a linear polarizer (LP) inserted between a quarter wave plate (QWP) and PD. The PD selectively detects the RCP component from the OBL2.

We measured the conversion efficiency by using the following equation:

$$\text{Conversion efficiency} = \frac{\text{Spin-converted output power}}{\text{Total output power}} = \frac{P_{\mathrm{SC}}/T_{\mathrm{LP}}}{P_{\mathrm{Total}}}. \tag{26}$$

Here, $P_{\mathrm{Total}}$ and $P_{\mathrm{SC}}$ indicate the powers measured by the photodiodes in the setups shown in Fig. S4a and b, respectively. $T_{\mathrm{LP}}$ is the transmission efficiency of the linear polarizer introduced to compensate for reduced intensity due to reflection or absorption.

While obtaining the conversion efficiency with respect to the numerical aperture (NA) of OBL 1, we set the NA of OBL2 to be similar to that of OBL1 to minimize the loss of transmission power after the propagation through the sample. The NAs of the OBL1 and OBL2 were (0.1/0.25), (0.25/0.28), (0.4/0.42), (0.55/0.42), (0.65/0.55), and (0.9/0.9), respectively.

## 3.2 Light source equipments

We applied a Coherent OBIS LS 594 nm 60 mW laser as the light source of the input beam shown in Fig. 3a for the hBN crystal-based vortex generator. Additionally, we used a 750 nm laser obtained by filtering the 10-nm-wide band from the SuperK Fianium supercontinuum laser source for the measurement of the conversion efficiency of $MoS_2$ crystal and flake. In addition, we used a 780 nm laser (Moglabs) to acquire the intensity profiles in Fig. S1 because it has a greater coherence length than the supercontinuum laser, enabling the creation of interference patterns.

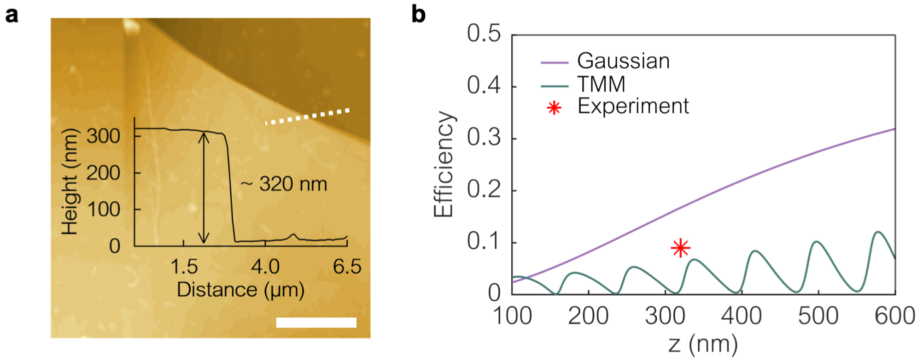


Figure S5: (a) An atomic force microscopy (AFM) profile of a $MoS_2$ flake on a glass substrate. The scale bar is 5 µm. The inset shows a one-dimensional height profile of the $MoS_2$ flake along the white dashed line in the image. (b) Calculated and experimental (red asterisk) conversion efficiencies with the $MoS_2$ flake. The calculated values by using Gaussian approximation and TMM methods are indicated by light purple and teal lines, respectively. The NA of the OBL1 is 0.9, and the wavelength of the input beam is 750 nm. $z$ corresponds to the propagation length and the flake thickness in the Gaussian beam approximation and TMM-based calculations, respectively.

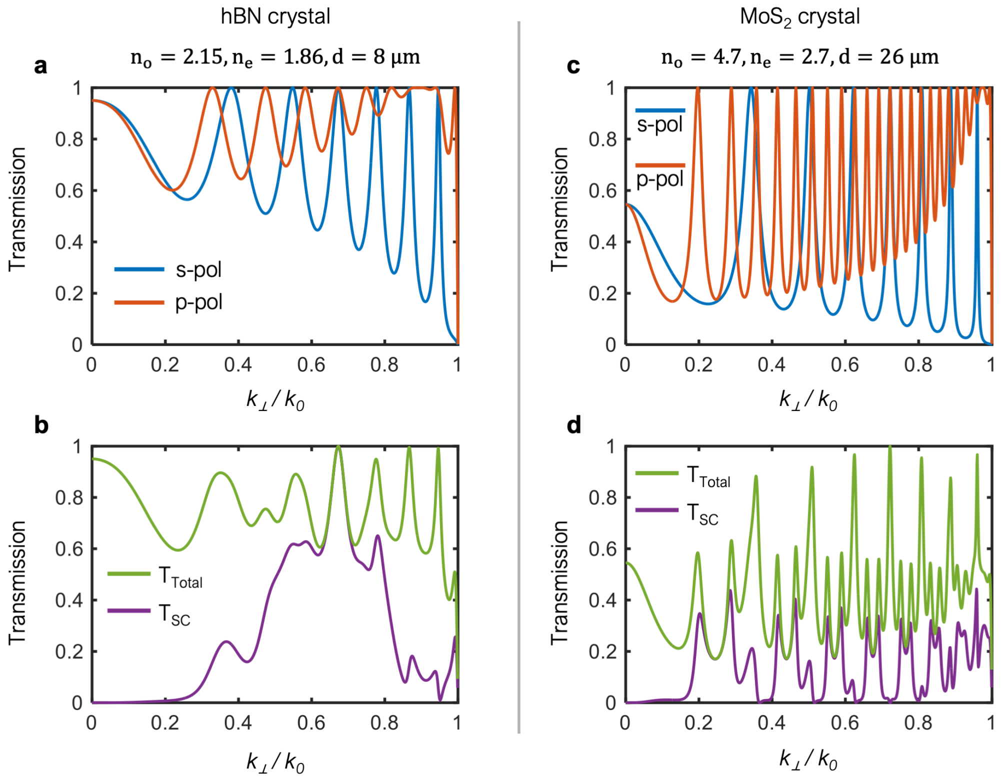


Figure S6: (a and c) Transmissions of 8-μm-thick hBN and 26-μm-thick $MoS_2$ crystals, respectively. The transmissions depend on the transverse wave-vector $k_\perp$ and vary with polarizations. The wavelengths for hBN and $MoS_2$ crystals are 594 and 750 nm, respectively. (b and d) Total and spin-converted transmissions ($T_{\mathrm{Total}}$ and $T_{\mathrm{SC}}$) of hBN and $MoS_2$ crystals, respectively. Note that the spin-converted transmission can reach near-unity at a specific point ($k_\perp/k_0 \sim 0.7$ at the hBN crystal).

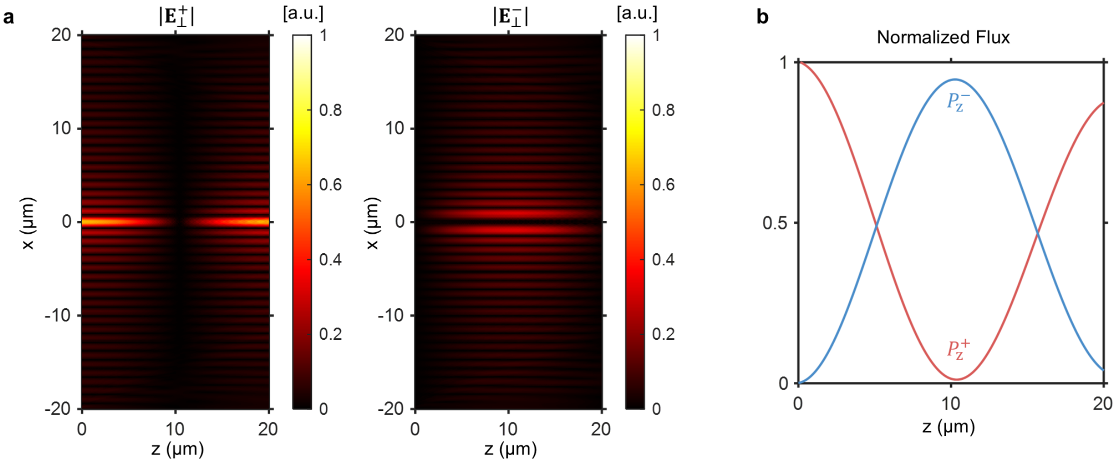


Figure S7: (a) Amplitude profile of a circularly polarized Bessel beam propagating along the extraordinary axis of the $MoS_2$ crystal. The incident beam's wavelength $\lambda$ is 750 nm. $|\mathbf{E}_\perp^+|$ and $|\mathbf{E}_\perp^-|$ indicate the absolute amplitudes of the LCP and RCP electric fields, respectively. Both amplitudes are normalized by the maximum value of themselves. (b) $z$-directional powers of the LCP ($P_z^+$) and RCP ($P_z^-$) waves.
